# Primary Care COPD Patients Compared with Large Pharmaceutically-Sponsored COPD Studies: An UNLOCK Validation Study

**DOI:** 10.1371/journal.pone.0090145

**Published:** 2014-03-05

**Authors:** Annemarije L. Kruis, Björn Ställberg, Rupert C. M. Jones, Ioanna G. Tsiligianni, Karin Lisspers, Thys van der Molen, Jan Willem H. Kocks, Niels H. Chavannes

**Affiliations:** 1 Department of Public Health and Primary Care, Leiden University Medical Centre, Leiden, The Netherlands; 2 Department of Public Health and Caring Science, Family Medicine and Preventive Medicine, Uppsala University, Uppsala, Sweden; 3 Peninsula Medical School, University of Plymouth, Plymouth, United Kingdom; 4 University of Groningen, University Medical Center Groningen, Department of General Practice, Groningen, The Netherlands; 5 University of Groningen, University Medical Center Groningen, GRIAC Research Institute, Groningen, The Netherlands; 6 Agia Barbara Health Care Centre, Heraklion, Crete, Greece; CUNY, United States of America

## Abstract

**Background:**

Guideline recommendations for chronic obstructive pulmonary disease (COPD) are based on the results of large pharmaceutically-sponsored COPD studies (LPCS). There is a paucity of data on disease characteristics at the primary care level, while the majority of COPD patients are treated in primary care.

**Objective:**

We aimed to evaluate the external validity of six LPCS (ISOLDE, TRISTAN, TORCH, UPLIFT, ECLIPSE, POET-COPD) on which current guidelines are based, in relation to primary care COPD patients, in order to inform future clinical practice guidelines and trials.

**Methods:**

Baseline data of seven primary care databases (n = 3508) from Europe were compared to baseline data of the LPCS. In addition, we examined the proportion of primary care patients eligible to participate in the LPCS, based on inclusion criteria.

**Results:**

Overall, patients included in the LPCS were younger (mean difference (MD)-2.4; p = 0.03), predominantly male (MD 12.4; p = 0.1) with worse lung function (FEV1% MD -16.4; p<0.01) and worse quality of life scores (SGRQ MD 15.8; p = 0.01). There were large differences in GOLD stage distribution compared to primary care patients. Mean exacerbation rates were higher in LPCS, with an overrepresentation of patients with ≥1 and ≥2 exacerbations, although results were not statistically significant. Our findings add to the literature, as we revealed hitherto unknown GOLD I exacerbation characteristics, showing 34% of mild patients had ≥1 exacerbations per year and 12% had ≥2 exacerbations per year. The proportion of primary care patients eligible for inclusion in LPCS ranged from 17% (TRISTAN) to 42% (ECLIPSE, UPLIFT).

**Conclusion:**

Primary care COPD patients stand out from patients enrolled in LPCS in terms of gender, lung function, quality of life and exacerbations. More research is needed to determine the effect of pharmacological treatment in mild to moderate patients. We encourage future guideline makers to involve primary care populations in their recommendations.

## Introduction

Chronic obstructive pulmonary disease (COPD) is one of the most complex diseases seen by respiratory physicians and general practitioners (GPs). Patients suffer from fluctuating episodes of exacerbations and airway symptoms which are difficult to control and may not sufficiently respond to inhalation therapy.

In the last 30 years, more than 50 (inter)national guidelines on the management of COPD have been published worldwide [1]. However, despite international dissemination and intensive promotion, guidelines are not widely adopted in daily practice [2]. Recently, two surveys revealed that COPD management by GPs was well below guideline-recommended levels, with many GPs having very limited knowledge of COPD and its management [3], [4]. Furthermore, about 25% of the GPs reported to be unfamiliar with GOLD and one-third with ATS/ERS guidelines [4]. Overall, non-guideline-informed management was a consequence of no availability, no confidence in gauging pharmacologic response, or because the GPs considered the guidelines too long, not relevant, or expressed no agreement with guidelines [3], [4].

Recommendations in guidelines are usually based on the strongest category of evidence: (meta-analyses of) randomized clinical trials (RCTs). These RCTs, particularly in medication studies, included large and selected COPD populations to ensure that the effect of the studied treatment is not concealed by confounding factors [5]. Furthermore, mild COPD patients are often neglected in these trials, as inclusion criteria are restricted to values of predicted forced-expiratory volume in 1 second (FEV_1_% predicted) below 70%. Moreover, selected patients are generally those with sufficient motivation and time to participate in a trial, and most likely to comply with medication and regular appointments. It is questionable whether the results of such RCTs can be extrapolated to all patients with COPD [5], [6]. However, reliable judgments about the external validity of RCTs are essential if treatments are to be used correctly in as many patients as possible in routine clinical practice [7]. Recent GOLD guidelines acknowledge this limited generalizability in COPD studies and state some considerations related to the results of these trials [8].

However, there still remains a paucity of data in the literature regarding COPD patient characteristics at the primary care level, and therefore it still remains unknown if there is overlap in disease characteristics of populations included in large RCT’s compared to the population seen in primary care. For example, exacerbation prevalence data in mild COPD patients remains still unknown, and exacerbation prevalence data in the other GOLD stages are solely based on the results of large trials [8].

To this end, the aim of this study was to evaluate the external validity of six large pharmaceutically sponsored COPD studies [9]–[14]. We aimed to provide insight into disease characteristics of COPD patients in primary care, in order to inform future guidelines and trialists. A secondary aim was to describe the proportion of primary care COPD patients eligible for inclusion in these studies.

## Materials and methods

### Study subjects

**UNLOCK patients:** Seven primary care databases from the UK, the Netherlands, Sweden and Greece were combined to create an extensive dataset of primary care COPD patients in the UNLOCK study [15]. All individual datasets included baseline data collected as part of on-going real-life cohort studies or pragmatic clinical trials in primary care. Inclusion criteria consisted of spirometrically validated COPD patients according to GOLD guidelines [8]; all studies applied few/limited or no exclusion criteria. Additional information on the methodology of the relevant studies is reported in the references. The UK dataset was a cohort study including 375 COPD diagnosed patients gathered to derive and validate a multicomponent assessment tool of COPD severity (the DOSE index); exclusion criteria consisted of serious co-morbidity affecting the patient’s ability to take part or to perform spirometry [16]. The Netherlands had four primary care datasets: two studies [one controlled clinical trial, Bocholtz study; n = 154 [17] and one cluster RCT, RECODE trial (Netherlands Trial Register (NTR) number 2268); n = 1086 [18]] aimed to evaluate the long-term effects of a multidisciplinary disease management program on quality of life. Both these studies included COPD patients and had limited exclusion criteria (terminal disease, immobility, substance abuse and inability to fill in questionnaires). The third dataset included 51 COPD diagnosed patients with a smoking history of > 10 years enrolled in a pilot for a RCT (The MARCH study; NTR number 2643) assessing the effect of health status guided care compared to GOLD guideline guided care in the primary care setting. [19] Exclusion criteria were patients with a myocardial infarction < 3 months ago, history of asthma/allergic rhinitis before age 40 years, oxygen use, dementia, or unstable or life-threatening comorbid condition. The fourth dataset comprised 1736 patients who were diagnosed and followed-up by the Asthma/COPD service in the Netherlands. This consultation service (including medical history, health status, lung function test and inhalation technique evaluation) is used by GPs for patients with (a suspicion of) asthma or COPD. For this latter study, only COPD patients were included and no exclusion criteria were applied. The Greek cohort study was designed to explore issues on quality of life, physical activity and dyspnea and included 109 primary care COPD patients with a smoking history of > 10 years; exclusion criteria were history of asthma, unstable cardiovascular disease, or any other respiratory disease other than COPD [20]. The dataset from Sweden included a cohort study (PRAXIS-study) of 775 primary care COPD patients aged 45–75 years, randomly selected from the medical records of 56 primary healthcare centres; there were no exclusion criteria [21], [22].

**Ethical approval:** The UK dataset was obtained with the aim to collect anonymised data on COPD patients. The South West Multicentre Research Ethics Committee confirmed that as a service evaluation, formal research ethics approval was not required for the audit. Patients were informed about the study and confidentiality issues. Patient consent was obtained to collect and analyse the data using an electronic consent form approved by the NHS information security and registration authority [16]. In the Dutch Bocholtz clinical trial, the regional Medical Ethics Committee of the Atrium Medical Centre, Heerlen, approved the study protocol. All patients gave written informed consent [17]. The Medical Ethics Committee of the Leiden University Medical Centre approved the Dutch RECODE trial, and all patients gave written informed consent [18]. Data from the Bocholtz and RECODE study is hosted at the department of Public Health and Primary Care at the Leiden University Medical Centre. The MARCH study was approved by the Medical Ethical Committee of the University Medical Centre Groningen, and data is hosted at the University Medical Centre Groningen. All patients gave written informed consent [19]. The fourth Dutch study consisted of observational, anonymised data from the large Asthma/COPD service in the Netherlands. The privacy regulation of the study was registered at the Dutch Data Protection Authority. According to current Dutch legislation, neither informed consent nor approval is required from a medical ethics committee for observational studies using anonymised data records [23]. The Greek study was approved by the local medical ethics committee of the University Hospital of Heraklion, Crete, Greece and all patients gave written informed consent. The data is hosted at the department of Thoracic Medicine in the University Hospital of Heraklion, Crete, Greece [20]. The Swedish study was approved by the Regional Ethical Review Board of Uppsala University, Uppsala, Sweden (Dnr 2004:M-445, Dnr 2010/090 and Dnr 2012/252). Written consent to use the information for future analysis was obtained for all participating patients in 2005. The data is hosted in the University of Uppsala, Sweden, at the department of Medical Sciences: Respiratory Medicine & Allergology [21], [22]. The first and last author received all anonymised study datasets and combined these in one dataset.

**Large pharmaceutically sponsored COPD studies (LPCS):** We compared the patient characteristics of the UNLOCK datasets with baseline data (if available) from six large pharmaceutically sponsored COPD studies (hereafter called the LPCS). These studies were published in the year 2000 or later: the ISOLDE [9], [24], [25], TRISTAN [10], TORCH [11], [26]–[29], UPLIFT [12], [30], ECLIPSE [13], [31], [32] and POET-COPD [14] studies. In addition to five large trials, we decided to include the ECLIPSE cohort study as well, because this is an important observational study often cited in guidelines, especially with regard to exacerbation frequency patterns.

### Outcomes

**Measurements:** Measurements included age, gender, smoking status, pack years, body mass index (BMI), lung function, dyspnea, health-related quality of life, and exacerbations. For smoking status, participants were categorized as never, ex- and current smokers and, if available, the number of pack years was calculated. BMI was calculated using [weight (kg)/(height (m)]^2^. In all patients, spirometry was performed according to international guidelines [8]. We grouped patients into GOLD stage categories based on their post-bronchodilator FEV_1_% predicted as follows: stage I corresponds to post-bronchodilator FEV_1_ ≥ 80% predicted, stage II to post-bronchodilator FEV_1_ 50% to < 80% predicted, stage III corresponds to 30% to <50% predicted, and stage IV corresponds to ≤ 30% predicted [8].

**Definition of exacerbations:** The definition of exacerbation used in the UNLOCK, ISOLDE [9], TRISTAN [10], TORCH [29] and ECLIPSE [32] studies was based on worsening of symptoms and the decision by a patient’s clinician (or by study personnel) to prescribe antibiotics or systemic corticosteroids, alone or in combination. In the UPLIFT [30] and POET-COPD [14] studies, an exacerbation was defined as an increase in or the onset of more than one respiratory symptom (cough, sputum, sputum purulence, wheezing, or dyspnea) lasting 3 days or more and requiring treatment with an antibiotic or a systemic corticosteroid. The mean exacerbation rate per person per year was calculated and subsequently distributed per GOLD stage. We also calculated the proportion of patients with at least one or two exacerbations per year and compared these to baseline values of the LPCS. If baseline data were missing, we contacted the authors of the studies to request more information. When we received no response or baseline data was not available, we used placebo-limb data of the LPCS. Additionally, we used recent data of the GOLD 2013 guidelines [8], in which reference values of exacerbation rates distributed per GOLD stage are stated.

**Dyspnea and health-related quality of life questionnaires:** Dyspnea was measured with the Medical Research Council (MRC) dyspnea score [33]. We used the St Georges Respiratory Questionnaire (SGRQ) which is designed to measure health- related quality of life in patients with asthma and COPD [34]. The Clinical COPD Questionnaire (CCQ) was also used: this is a disease-specific 10-item questionnaire that calculates an overall score and three domain scores: symptoms, functional state and emotional state; patients are required to respond to each item on a 7-point scale with 0 representing the best possible score and 6 representing the worst possible score [35].

### Data acquisition

Data on age, gender, lung function and GOLD stage were available for all patients. The UNLOCK datasets had additional data on the following subsets: current smoke status (98%), CCQ (98%), number of exacerbations (79%), BMI (61%), MRC dyspnea score (41%), SGRQ (32%) and pack years (25%). Mean exacerbation rates in the UNLOCK study were calculated using the number of exacerbations per patient in the year prior to inclusion in the study, divided by the total number of patients in the dataset, which provided data on the number of exacerbations.

### Data analysis

All analyses were performed with SPSS software, version 21. There were seven UNLOCK datasets. We calculated proportions for frequencies, and means for continuous variables, for every individual UNLOCK dataset. We used the means of the LPCS reported in the original publications as a comparison. Using independent sample t-tests, we tested the means of the seven (or less, in case of subsets of data) UNLOCK studies to the means of the six LPCS and reported mean differences, 95% confidence intervals (CI) and p-values. We performed a sensitivity analysis on primary care patients with GOLD stage II or above, in order to compare whether patients enrolled in trials were similar to the more severe patients in the primary care setting.

Furthermore, step-by-step we applied the inclusion criteria of the trials [9], [10], [14], [29], [30], [32] to the UNLOCK population and calculated the proportion of patients eligible for inclusion.

## Results

### Baseline comparisons

**Individual datasets:** The UNLOCK datasets included a total of 4286 patients diagnosed with COPD by a GP or respiratory physician. After exclusion of patients with missing lung function data (N = 524; 12%) and a ratio of FEV_1_/FVC of ≥ 0.7 (N = 254; 7%), baseline characteristics of the remaining 3508 primary care COPD patients were compared with those of the LPCS. Results of baseline characteristics of the individual UNLOCK datasets and the LPCS are reported in Table 1.

**Table 1:** Descriptive baseline data of the seven primary care UNLOCK datasets, compared with baseline data of six large pharmaceutically sponsored COPD studies.

Characteristic	UNLOCK 1 NL	UNLOCK 2 UK	UNLOCK 3 NL	UNLOCK 4 NL	UNLOCK 5 GR	UNLOCK 6 SW	UNLOCK 7 NL	ISOLDE 2000	TRISTAN 2003	TORCH 2007	UPLIFT 2008	ECLIPSE 2010	POET-COPD 2011
**Patients (N)**	86	375	1665	51	96	333	902	751	1465	6112	5992	2164	7376
**Age, years**	65.1 (9.9)	69.2 (8.6)	66.8 (10.7)	63.6 (10.5)	66.3 (8.7)	63.3 (8)	68.5 (10.9)	63.7 (7.1)	62.7 (8.7)	65.0 (8.3)	64.5 (8.4)	63.4 (7.1)	62.9 (9.0)
**Male, % (n/N)**	70 (60/86)	60 (223/375)	59 (976/1665)	61 (31/51)	92 (88/96)	41 (137/333)	45 (407/902)	75	75	76	75	65	74
**Current smokers, % (n/N)**	42 (36/86)	32 (120/375)	50 (826/1665)	57 (29/51)	50 (48/96)	35 (117/333)	37 (313/855)	36	52	43	29	36	48
**Pack years**	35.1 (22)	45.1 (27)	-	39.5 (17.9)	66 (33.2)	-	32 (26.5)	44 (30)	42.0 (22.4)	47.0 (26.5)	49.0 (28.0)	48.6 (27.1)	38.8 (20.0)
**BMI, kg/m^2^**	25.8 (5.4)	26.7 (5.6)	26.4 (4.6)	-	-	-	-	24.5 (4.8)	-	25.4 (5.2)	26.0 (5.1)	26.5 (5.7)	-
**Postbronchodilator FEV_1_ (% predicted)**	62.9 (19)	49.9 (14.4)	68 (17.9)	75.8 (16)	55.3 (18.7)	68.9 (23.9)	65.9 (19.9)	50.3 (14.9)	44.8 (14.7)*	44.3 (12.3)	47.7 (12.7)	48.3 (15.8)	49.2 (13.3)
**FEV1:FVC (%)**	59.4 (10.2)	-	55 (10.8)	-	56.1 (10.4)	-	55.2 (11.7)	43 (12)	-	48.7 (10.8)*	43.6 (10.8)	44.8 (11.6)	52.5 (10.8)
**GOLD distribution**													
Mild GOLD I, %	20 (17/86)	-	26 (440/1665)	35 (18/51)	6 (6/96)	33 (111/333)	25 (232/902)	0	-	0	0	0	0
Moderate GOLD II, %	50 (43/86)	52 (196/375)	57 (943/1665)	59 (30/51)	60 (58/96)	42 (139/333)	53 (477/902)	52	-	35	46	44	48
Severe GOLD III, %	27 (23/86)	39 (145/375)	16 (263/1665)	6 (3/51)	19 (18/96)	21 (71/333)	19 (168/902)	III & IV: 48	-	49	44	42	43
Very severe GOLD IV, %	3 (3/86)	9 (34/375)	1 (19/1665)	-	15 (14/96)	4 (12/333)	3 (25/902)		-	15	8	14	9
**Patient-reported outcomes**													
SGRQ score	33.4 (20.5)	-	-	23.6 (14.8)	37.5 (20)	-	35.8 (20.3)	49.9 (17.4)	47.1 (15.7)	49.3 (17.1)	45.7 (17.0)	50.1 (20.3)	-
CCQ score	1.5 (1.0)	2.0 (1.1)	1.5 (1.0)	1.1 (0.8)	1.6 (0.9)	1.9 (1.2)	1.5 (1.0)	-	-	-	-	-	-
MRC score	2.3 (1.1)	2.7 (1.0)	-	0.7 (0.8)	2.0 (1.0)	2.8 (1.4)	2 (1.3)	-	-	-	-	2.7 (1.1)	-
MRC score > 2, % (n/N)	35 (30/86)	49 (164/337)	-	2 (1/49)	27 (26/96)	47 (152/324)	34 (303/898)	-	-	50	-	53	-

Results are means, unless otherwise noted. “-“ indicates data not available. *indicates Pre-bronchodilator values.

Note: In TORCH, UPLIFT, POET-COPD, TRISTAN mean data for total group were not published, we used the following baseline data: POET-COPD: Tiotropium group; UPLIFT: Tiotropium group; ECLIPSE: total group; TORCH: combination therapy group; TRISTAN: Salmeterol/Fluticasone group; ISOLDE: Fluticasone group.

Abbreviations: NL: Netherlands; UK: United Kingdom; GR: Greece; SW: Sweden; BMI: body mass index; FEV1: forced expiratory volume in 1 second; GOLD: Global initiative for chronic Obstructive Lung Disease; SGRQ: St Georges Respiratory Questionnaire; CCQ: Clinical COPD Questionnaire; MRC: Medical Research Counsil.

**Overall means of the UNLOCK and LPCS studies:** The overall means of the UNLOCK studies and the LPCS, including the results of the independent sample t-tests, are reported in Table 2. Compared with the UNLOCK studies, the LPCS included a statistically significant (mean difference (MD) -2.4; p = 0.03) younger population with a higher proportion of males (MD 12.4; p = 0.1) and significant lower FEV_1_% predicted values (MD -16.4; p<0.01) and lower FEV1/FVC values (MD -9.2; p<0.01). There were large differences in GOLD distribution between the UNLOCK studies and the LPCS. There was total absence of GOLD I in the LPCS, whilst in the UNLOCK studies, mild and moderate patients (GOLD I and II) comprised 74% of the total COPD population. In the LPCS, the proportion of GOLD III patients were more than doubled compared to the UNLOCK population (44.5% versus 21%, MD 23.5; p<0.01). In addition, primary care patients in the UNLOCK studies had significantly better health-related quality of life (measured with the SGRQ) compared with LPCS (MD 15.8; p = 0.01). In the TORCH and ECLIPSE studies, the proportion of patients with an MRC score >2 was measured, and in ECLIPSE the mean MRC scores were reported as well. Overall mean MRC scores were similar in the UNLOCK studies compared to ECLIPSE: 2.1 (0.8) and 2.7 (1.1), respectively. However, overall 51.5% of the patients in the ECLIPSE and TORCH studies had an MRC score >2, meaning walking slower than most people on the level, whereas in the UNLOCK studies this overall proportion was 32.3%, this mean difference was statistically significant (p = 0.04).

**Table 2:** Baseline comparison of the UNLOCK studies versus large COPD studies, including independent sample t-tests.

Characteristic	UNLOCK studies	Large COPD studies (LPCS)	Mean difference between UNLOCK – LPCS (95% CI)	p-value
**Patients (N)**	3508	23860		
**Age, years**	66.1 (2.3)	63.7 (0.9)	–2.4 (–4.6 — –0.3)	0.03*
**Male, %**	60.9 (16.7)	73.3 (4.1)	12.4 (–3.1—27.9)	0.1
**Current smokers, %**	42.9 (9.5)	40.7 (8.6)	–2.2 (–13.2—8.8)	0.67
**Pack years**	43.6 (13.5)	44.9 (4.03)	1.3 (–15.2—17.8)	0.84
**BMI, kg/m^2^**	26.3 (0.5)	25.6 (0.9)	–0.7 (–2 —0.6)	0.23
**Postbronchodilator FEV_1,_ % predicted**	63.8 (8.7)	47.4 (2.4)	–16.4 (–24—–8.2)	<0.01*
**FEV1:FVC, %**	55.7 (0.7)	46.5 (4.0)	–9.2 (–14.1 —–4.2)	<0.01*
**GOLD distribution**				
Mild GOLD I	20.7 (13.2)	-	-	-
Moderate GOLD II	53.3 (6.2)	45 (6.3)	–8.3 (–16.6—0.1)	0.05
Severe GOLD III	21 (10.1)	44.5 (3.1)	23.5 (13.9—33.1)	<0.01*
Very severe GOLD IV	5.8 (5.2)	11.5 (3.5)	5.7 (–0.71—12)	0.08
**Patient-reported outcomes**				
SGRQ	32.6 (6.2)	48.4 (1.9)	15.8 (6.3—25.4)	0.01*
CCQ (mean)	1.6 (0.3)	-	-	-
MRC (mean)	2.1 (0.8)	2.7 (1.1)	0.6 (–1.5—2.7)	0.5
MRC score > 2 (%)	32.3 (17)	51.5 (2.1)	19.2 (1.3—37)	0.04*

Data are overall mean values (SD), in which every dataset or study contributed equally to the overall means. “-“ indicates data not available. 95% CI: 95% confidence interval. Abbreviations: BMI: body mass index; FEV1: forced expiratory volume in 1 second; GOLD: Global initiative for chronic Obstructive Lung Disease; SGRQ: St Georges Respiratory Questionnaire; CCQ: Clinical COPD Questionnaire; MRC: Medical Research Counsil.

### Exacerbation data

**Individual datasets:** UNLOCK studies reporting exacerbation data were compared with baseline data of the ISOLDE, TRISTAN, TORCH, UPLIFT and ECLIPSE studies (Table 3). There was heterogeneity between UNLOCK studies, with studies from the Netherlands reporting lower exacerbation rates compared to the UK study.

**Table 3:** Exacerbation data of the UNLOCK studies, compared with exacerbation data of the large pharmaceutically sponsored COPD studies.

Characteristic	UNLOCK 1 NL	UNLOCK 2 UK	UNLOCK 3 NL	UNLOCK 7 NL	ISOLDE 2000	TRISTAN 2003	TORCH 2007	UPLIFT 2008	ECLIPSE 2010
**Patients (N)**	86	375	1665	902	370 *	361 *	6112	5992	2138
Mean exacerbation rate p/year	1.05 (1.3)	1.32 (1.6)	0.72 (1.1)	0.54 (1.19)	1.90 (2.63)*	1.30 *	1.0 (1.3)	0.85 (0.02)*	0.9 (1.2)
≥1 in preceding year, % (n/N)	55 (47/85)	59 (222/374)	43 (713/1661)	27 (174/636)	63*	-	57	68*	47
≥2 in preceding year, % (n,N)	29 (25/85)	33 (124/374)	19 (312/1661)	11 (72/636)	-	-	32	-	29

Data are baseline data and mean values (SD), unless stated otherwise. *indicates data from placebo group; “-“ indicates data not available.

**Overall means of the UNLOCK and LPCS studies:** The UNLOCK studies reported a lower mean exacerbation rate per year compared to the LPCS (MD 0.3; p = 0.31), as well as a lower proportion of patients with ≥ 1 (MD 15; p = 0.21) or ≥ 2 exacerbations (MD 8; p = 0.24); Table 4.

**Table 4:** Mean exacerbation data of the UNLOCK studies, compared with mean exacerbation data of the large pharmaceutically sponsored COPD studies, including independent sample t-tests.

Characteristic	UNLOCK studies	Large COPD studies (LPCS)	Mean difference between UNLOCK-LPCS (95% CI)	p-value
**Patients (N)**	3028	14973		
Mean exacerbation rate p/year	0.9 (3.5)	1.2 (0.4)	0.3 (–0.3—0.9)	0.31
Mean % of patients with ≥1 exacerbation in preceding year	44 (14.4)	59 (9)	15 (–12—42)	0.21
Mean % of patients with ≥2 in preceding year	22 (10)	30 (2.1)	8 (–9—25)	0.24

Data are overall mean values (SD), in which every dataset or study contributed equally to the overall means. 95% CI: 95% confidence interval.

### Exacerbation data distributed per GOLD stage

Exacerbation characteristics distributed per GOLD stage are shown in Table 5 (individual datasets) and Table 6 (overall means). When the severity of COPD increased (as measured with GOLD), the proportion of COPD patients with at least one or two exacerbations also increased, the exception being patients in GOLD stage IV in UNLOCK patients, which had a lower proportion compared to GOLD III on all these variables. Furthermore, differences between GOLD stages in patients with ≥ 1 or ≥ 2 exacerbation in the UNLOCK studies were not as high as reported in the LPCS (Table 6).

**Table 5:** Exacerbation characteristics distributed per GOLD stages: large COPD studies, compared to UNLOCK primary care datasets.

Characteristic	UNLOCK 1 NL	UNLOCK 2 UK	UNLOCK 3 NL	UNLOCK 7 NL	ISOLDE 2000	TRISTAN 2003	TORCH 2007	UPLIFT 2008	ECLIPSE 2010	GOLD GUIDELINE 2013
**Patients (N)**	86	375	1665	902	370 *	361 *	6112	5992	2138	
**≥1 exacerbations p/year distributed per GOLD stage**										
GOLD I, % (n/N)	47 (8/17)	-	32 (140/438)	24 (38/160)	-	-	-	-	-	-
GOLD II, % (n/N)	44 (19/43)	58 (114/196)	46 (435/941)	24 (83/346)	71*	-	-	65*	29	-
GOLD III, % (n/N)	82 (18/22)	60 (86/143)	49 (128/263)	42 (47/113)	GOLD III&IV: 84*	-	-	71*	52	-
GOLD IV, % (n/N)	67 (2/3)	62 (21/34)	53 (10/19)	35 (6/17)		-	-	69*	62	-
**≥2 exacerbations p/y distributed per GOLD stage**										
GOLD I, % (n/N)	12 (2/17)	-	14 (60/438)	10 (16/160)	-	-	-	-	-	-
GOLD II, % (n/N)	26 (11/43)	30 (59/196)	21 (194/941)	8 (28/346)	-	-	-	-	22	-
GOLD III, % (n/N)	50 (11/22)	36 (51/143)	21 (55/263)	21 (24/113)	-	-	-	-	33	-
GOLD IV, % (n/N)	33 (1/3)	38 (13/34)	16 (3/19)	24 (4/17)	-	-	-	-	47	-
**Exacerbation rate distributed per GOLD stage**										
GOLD I	0.65	-	0.53	0.46	-	-	-	-	-	-
GOLD II	0.81	1.23	0.78	0.41	0.92*	-	0.9	0.70*	0.85	0.7–0.9
GOLD III	1.82	1.3	0.86	1	III&IV: 1.75*	-	1.0	0.97*	1.34	1.1–1.3
GOLD IV	1	1.79	0.74	1		-	1.3	1.15*	2.00	1.2–2.0

Data are proportions and mean values (SD). “-“ indicates data not available. * indicates data from placebo group.

GOLD guideline data was reported in the GOLD guidelines of 2013 [8] and was based on the placebo-limbed data of the TORCH, UPLIFT and ECLIPSE studies.

Abbreviations: GOLD: Global initiative for chronic Obstructive Lung Disease.

**Table 6:** Exacerbation characteristics distributed per GOLD stages: means of the large COPD studies, compared to mean of the UNLOCK studies

Characteristic	UNLOCK studies	Large COPD studies (LPCS)	Mean difference between UNLOCK-LPCS (95% CI)	p-value
**Patients (N)**	3028	14973		
**≥1 exacerbations p/year distributed per GOLD stage**				
GOLD I, % (n/N)	34.3 (11.7)	-	-	
GOLD II, % (n/N)	43 (14.1)	58.3 (17)	15.3 (–18.6—49.2)	0.28
GOLD III, % (n/N)	58.3 (17.5)	69 (16.1)	10.7 (–22.8—44.3)	0.33
GOLD IV, % (n/N)	54.3 (14.1)	71.7 (11.2)	17.4 (–7.3—42.1)	0.13
**≥2 exacerbations p/y distributed per GOLD stage**				
GOLD I, % (n/N)	12 (2.0)	-	-	
GOLD II, % (n/N)	21.3 (9.6)	22	15.3 (–18.6—49.2)	0.28
GOLD III, % (n/N)	32 (13.9)	33	10.7 (–22.8—44.3)	0.33
GOLD IV, % (n/N)	27.8 (9.7)	47	17.4 (–7.3—42.1)	0.13
**Exacerbation rate distributed per GOLD stage**				
GOLD I	0.55 (1)	-	-	
GOLD II	0.81 (0.3)	0.84 (1)	0.03 (–0.5—0.5)	0.85
GOLD III	1.25 (0.4)	1.27 (0.4)	0.02 (–0.7—0.7)	0.95
GOLD IV	1.13 (0.5)	1.55 (0.4)	0.42 (–0.32—1.16)	0.22

Data are overall mean values (SD), in which every dataset or study contributed equally to the overall means. “-“ indicates data not available. 95%CI : 95% confidence interval. Abbreviations: GOLD: Global initiative for chronic Obstructive Lung Disease.

### Sensitivity analysis

We performed a sensitivity analysis on the UNLOCK datasets including only patients with GOLD stage II or above, in order to compare whether patients enrolled in the trials are similar to the more severe patients in primary care. There was no difference between the sensitivity analysis (Table S1) and Tables 2 and 4.

### Selection for large COPD studies

The proportion of patients from primary care that would be eligible to be included in the LPCS ranged from 17% (TRISTAN trial) to 42% (ECLIPSE and UPLIFT study) (Table 7). The LPCS inclusion criteria of at least one exacerbation in the preceding year and an FEV_1_ of < 60% predicted, excluded the largest proportion of primary care patients, as only 44% (≥ 1 exacerbation in previous year) and 39.3% (FEV_1_≤ 60% predicted) of the patients, respectively, fulfilled these criteria.

**Table 7:** Percentage of patients remaining after introduction of different selection criteria used in six large COPD studies.

**Age**	**40**–**75 years**		**40**–**80 years**	**≥ 40 years**	**40**–**75 years**	**≥ 40 years**
% of patients in primary care	77.2		89.9	99.7	77.2	99.7
**FER**	**≤ 70%**	**≤ 70%**	**≤ 70%**	**≤ 70%**	**≤ 70%**	**≤ 70%**
% of patients in primary care	93.2	93.2	93.2	93.2	93.2	93.2
**FEV_1_**	**< 85%**	**25**–**70%**	**< 60%**	**≤ 70%**	**< 80%**	**≤ 70%**
% of patients in primary care	83.4	57.9	39.3	59.3	76.5	59.3
**Reversibility**	**≤ 10%**	**≤ 10%**	**≤ 10%**			
% of patients in primary care	78.4	78.4	78.4			
**Smoking status**	**Current/ex-smoker**		**Current/ex-smoker**	**Current/ex-smoker**	**Current/ex-smoker**	
% of patients in primary care	82.3		82.3	82.3	82.3	
**Pack years**		**≥ 10 pack years**	**≥ 10 pack years**	**≥ 10 pack years**	**≥ 10 pack years**	**≥ 10 pack years**
% of patients in primary care		93	93	93	93	93
**Exacerbation**		**≥ 1 exacerbation in previous year**				**≥ 1 exacerbation in previous year**
		44				44
**Total % of patients in primary care**	**39**	**17**	**20**	**42**	**42**	**23**

## Discussion

This is the first study using a large international COPD primary care dataset from Europe to compare disease characteristics of primary care COPD patients with disease characteristics of COPD populations included in large pharmaceutically-sponsored COPD studies (the LPCS). We demonstrated there were clear differences in gender, age, distribution of GOLD stages, quality of life scores and exacerbation characteristics between COPD patients seen in primary care and included in the LPCS. As a result, the majority (58–83%) of COPD patients in primary care would not serve as a candidate for inclusion in these LPCS.

The present study provides insight into the disease characteristics of COPD in primary care, including the milder affected patients. According to GOLD guidelines [8], prevalence data on exacerbation rates in GOLD I were currently lacking in literature, whereas data on GOLD II-IV stages were based on selected COPD populations of the LPCS and were not validated in primary care. In the ECLIPSE study, Hurst et al. showed that the best predictor of exacerbations (across all GOLD stages) was an exacerbation history [13]. Interestingly, we determined that 12% of GOLD I patients in primary care were frequent exacerbators (≥ 2 exacerbations per year). Furthermore, we demonstrated 34% of GOLD I patients exacerbated at least once yearly. Since moderate COPD is more prevalent than very severe COPD, the overall burden of exacerbations in terms of FEV_1_ decline, and the costs may be greater with milder disease [13]. On the other hand, physicians should be aware that the majority of mild COPD patients are often symptom free and often remain undiagnosed, as earlier demonstrated in the international BOLD and PLATINO studies [36], [37]. It could be important to intervene at an early stage of the disease [38], but pharmacological intervention should in general be reserved for symptomatic patients or frequent exacerbators, whilst asymptomatic GOLD I patients can be offered non-pharmacological strategies, such as smoking cessation, aimed at preventing further worsening of the disease.

Interestingly, we demonstrated the majority of COPD patients in primary care would not serve as a candidate for inclusion in large pharmaceutically sponsored studies. As a result, primary care physicians are left to treat patients based on results derived from trials that their patients would not have been eligible to join. The economic impact of this low external validity potentially leads to considerable avoidable costs. Over-prescribing of inhaled steroids in primary care is described in various countries [16], [39]–[42]. One recent UK primary care study concluded 38% of patients were over-treated regarding their GOLD stage, with considerable potential for harm and a mean extra per patient cost of £553.56/year [41]. This is in line with results of a Spanish primary care study, in which 18.2% of patients received inhalation therapy not meeting criteria for its use as recommended in guidelines, which was associated with lower physical health status and higher annual costs [42]. The revised GOLD 2013 guidelines acknowledge the lack of evidence concerning anti-inflammatory and bronchodilator medications in patients with GOLD stage I and II [8]. Subsequent post-hoc analysis of the TORCH trial concluded that a combination of salmeterol and fluticasone propionate reduced exacerbations and FEV_1_ decline in patients with a FEV_1_ of 50–60% predicted [28]. However, that study was not specifically powered to show differences between GOLD stages and, as inclusion was restricted to FEV_1_ of 60%, many GOLD II patients were not included. Subgroup analysis of UPLIFT showed promising results of tiotropium in GOLD II patients on FEV_1_ decline [12], but inclusion was limited to patients with FEV1<70%, leading to incomplete representation of GOLD II. More research is needed to determine the effect of inhalation therapies in mild to moderate COPD patients, and we strongly encourage guideline makers to base their recommendations on primary care studies as well.

Our population based data reflect the recent tendency towards an increasing prevalence of COPD in women, drawing a different picture of the current COPD patient than the one represented in the LPCS. In fact, underrepresentation of women in large medical trials is not uncommon, resulting in a call in Nature for other journals, funding agencies and researchers to give women parity with men [43]. As the prevalence of COPD in women is rising, we advise future trialists to include not only milder COPD patients, but also more female participants, in order to study whether biological differences affect the way women respond to medications and therapeutic strategies.

Two studies published in 2005 and 2007 evaluated the external validity in COPD patients using smaller datasets [5], [6]. Although their results are in line with our conclusions, there are some important differences. Herland et al. used a population of mixed obstructive lung disease and concluded that only 17% of these patients were eligible for inclusion in a typical COPD RCT; included COPD patients (n = 366) were not classified according to GOLD criteria, but were graded using a 10-cm free-graded visual analogue scale which had to differentiate between asthma, COPD and mixed obstructive lung disease [5]. In the study of Travers et al., only 0–9% of COPD patients were eligible for inclusion based on the very strict criteria of trials conducted between 1994 and 2003 [6]. It is likely that in more recent years less strict inclusion criteria were used for participation in a trial.

This study has several limitations that should be addressed. First, the current analysis of primary care COPD patients was restricted to seven datasets from four different countries in Europe; as a consequence, our data will probably not be representative for all primary care populations worldwide. Second, as there are differences in COPD patients between countries, there was considerable heterogeneity between populations in the different databases, with for example patients from the UK having more exacerbations per year and worse quality of life scores compared to Dutch patients. Although it would be interesting to further evaluate these differences between countries, the present study does not allow drawing firm conclusions about these differences. Perhaps this study will provide an useful starting point for further validation in a larger, more diverse population of COPD patients across a multitude of different countries. Third, all individual datasets included baseline data collected in different designs of studies ranging from pragmatic clinical trials to real-life cohort studies. Irrespective of these varying designs, all studies had few or no exclusion criteria, making the dataset a reasonably representative sample of primary care populations in these countries. Fourth, as data were accessed retrospectively from different types of studies, some data were available on subsets of outcomes. As a result of heterogeneity and a low number of studies used for independent sample t-tests, on some outcomes mean differences were large and represented important findings, whilst showing no statistically significance. Therefore, the statistical tests performed in this study should be interpreted with caution. However, our aim was to provide illustrative findings rather than to be conclusive, and we assumed that our findings are based on a representative sample of primary care patients. In addition, we feel we provided an overall dataset large enough to make reliable comparisons with the LPCS, as we evaluated a similar number of included patients. Finally, another limitation is that, for comparison purposes, the present study compared primary care data to six LPCS. Although many other large COPD studies have been published over the years, we chose to evaluate the studies most frequently referred to in the guidelines, and published in the last decade.

## Conclusion

This study provides an informative insight into COPD patient characteristics in primary care. Overall, compared to primary care patients, patients in large pharmaceutically sponsored trials were younger, predominantly male with worse lung function and worse quality of life scores. Our findings add to the literature, as we revealed hitherto unknown GOLD I exacerbation characteristics, showing 34% of mild patients had ≥1 exacerbations per year and 12% had ≥ 2 exacerbations per year. Additionally, the majority of patients seen in primary care would not be eligible to be included a large pharmaceutically sponsored trial. Therefore, more research is needed to determine the effect of pharmacological treatment in mild to moderate patients. Furthermore, we encourage future guideline makers to involve primary care populations in their recommendations as well.

## Supporting Information

Table S1 **Sensitivity analysis on UNLOCK patients with GOLD stage II or above; comparison with large COPD studies, including independent sample t-tests.**
(DOCX)
