# Correction: Primary Care COPD Patients Compared with Large Pharmaceutically-Sponsored COPD Studies: An UNLOCK Validation Study

**DOI:** 10.1371/journal.pone.0098725

**Published:** 2014-05-23

**Authors:** The PLOS ONE Staff

There is an error in Table 7. The top row for this table is missing. Please view the correct Table 7 here:

**Table 7:** Percentage of patients remaining after introduction of different selection criteria used in six large COPD studies.

Inclusion criteria	ISOLDE 2000	TRISTAN 2003	TORCH 2007	UPLIFT 2008	ECLIPSE 2010	POET-COPD 2011
**Age**	**40-75 years**	**-**	**40-80 years**	**≥40 years**	**40-75 years**	**≥40 years**
% of patients in primary care	77.2		89.9	99.7	77.2	99.7
**FER**	**≤70%**	**≤70%**	**≤70%**	**≤70%**	**≤70%**	**≤70%**
% of patients in primary care	93.2	93.2	93.2	93.2	93.2	93.2
**FEV_1_**	**<85%**	**25-70%**	**<60%**	**≤70%**	**<80%**	**≤70%**
% of patients in primary care	83.4	57.9	39.3	59.3	76.5	59.3
**Reversibility**	**≤10%**	**≤10%**	**≤10%**	-	-	-
% of patients in primary care	78.4	78.4	78.4			
**Smoking status**	**Current/ex-smoker**	**-**	**Current/ex-smoker**	**Current/ex-smoker**	**Current/ex-smoker**	**-**
% of patients in primary care	82.3		82.3	82.3	82.3	
**Pack years**	**-**	**≥10 pack years**	**≥10 pack years**	**≥10 pack years**	**≥10 pack years**	**≥10 pack years**
% of patients in primary care		93	93	93	93	93
**Exacerbation**	**-**	**≥1 exacerbation in previous year**	**-**	**-**	**-**	**≥1 exacerbation in previous year**
		44				44
**Total % of patients in primary care**	**39**	**17**	**20**	**42**	**42**	**23**
