# Supplementary material for: Primary Care COPD Patients Compared with Large Pharmaceutically-Sponsored COPD Studies: An UNLOCK Validation Study
Source: PLoS One. 2014 Mar 5;9(3):e90145. doi: 10.1371/journal.pone.0090145 (PMC3943905; doi:10.1371/journal.pone.0090145)
Supplement: Table S1 — Sensitivity analysis on UNLOCK patients with GOLD stage II or above; comparison with large COPD studies, including independent sample t-tests. (DOCX) [file pone.0090145.s001.docx]

Supporting information

| **Characteristic** | **UNLOCK studies** | **Large COPD studies (LPCS)** | **Mean difference between**  **UNLOCK – LPCS (95% CI)** | **p-value** |
| --- | --- | --- | --- | --- |
| **Patients (N)** | 3508 | 23860 |  |  |
| **Age, years** | 66.3 (2.3) | 63.7 (0.9) | -2.6 (-4.8—-0.4) | 0.03* |
| **Male, %** | 59.6 (16.2) | 73.3 (4.1) | 13.7 (-1.3—28.8) | 0.07 |
| **Current smokers, %** | 42.4 (9.1) | 40.7 (8.6) | -1.7 (4.9—-12.5) | 0.73 |
| **Pack years** | 43.6 (13.5) | 44.9 (4) | 1.3 (-15.2—17.8) | 0.84 |
| **BMI, kg/m^2^** | 26.2 (0.5) | 25.6 (0.9) | -0.6 (-2—0.7) | 0.28 |
| **Postbronchodilator FEV_1,_ % predicted** | 57 (5.4) | 47.4 (2.4) | -9.6 (-14.8— -4.4) | <0.01* |
| **FEV1:FVC, %** | 53.5 (1.6) | 46.5 (4) | -7 (-11.9—-2.1) | 0.01* |
| **GOLD distribution** |  |  |  |  |
| Moderate GOLD II | 68.7 (12.5) | 45 (6.3) | -23.7 (-36—11.3) | <0.01* |
| Severe GOLD III | 25.6 (44.5) | 44.5 (3.1) | 18.9 (9.5—28.2) | <0.01* |
| Very severe GOLD IV | 6.6 (5) | 11.5 (3.5) | 4.9 (-1.4—11.1) | 0.11 |
| **Patient-reported outcomes** |  |  |  |  |
| SGRQ | 35.1 (5.8) | 48.4 (1.9) | 13.3 (4.5—22.2) | 0.02* |
| CCQ (mean) | 1.7 (0.3) | - |  |  |
| MRC (mean) | 2.1 (0.8) | 2.7 | 0.6 (-1.8—3) | 0.54 |
| MRC score > 2 (%) | 32.3 (18.9) | 51.5 (2.1) | 19.2 (-4.11—42.4) | 0.09 |
| **Exacerbation-related outcomes** |  |  |  |  |
| Mean exacerbation rate p/yr | 1.0 (0.3) | 1.2 (0.4) | 0.2 (-0.4—0.8) | 0.39 |
| ≥1 exacerbation in preceding yr | 48 (14) | 58.8 (9) | 10.7 (-10.6—32) | 0.25 |
| ≥2 exacerbation in preceding yr | 24.8 (10.6) | 30.5 (2.1) | 5.7 (-10.6—22) | 0.37 |

Table S1. Sensitivity analysis on UNLOCK patients with GOLD stage II or above; comparison with large COPD studies, including independent sample t-tests.

Results are means (SD). Abbreviations: BMI: body mass index; FEV1: forced expiratory volume in 1 second; FVC: Forced vital capacity; GOLD: Global initiative for chronic Obstructive Lung Disease; SGRQ: St Georges Respiratory Questionnaire; CCQ: Clinical COPD Questionnaire; MRC: Medical Research Counsil
